# Supplementary material for: Microbial growth under drought is confined to distinct taxa and modified by potential future climate conditions
Source: Nat Commun. 2023 Sep 22;14:5895. doi: 10.1038/s41467-023-41524-y (PMC10516970; doi:10.1038/s41467-023-41524-y)
Supplement: Supplementary file 1 — Supplementary Information [file 41467_2023_41524_MOESM1_ESM.pdf]

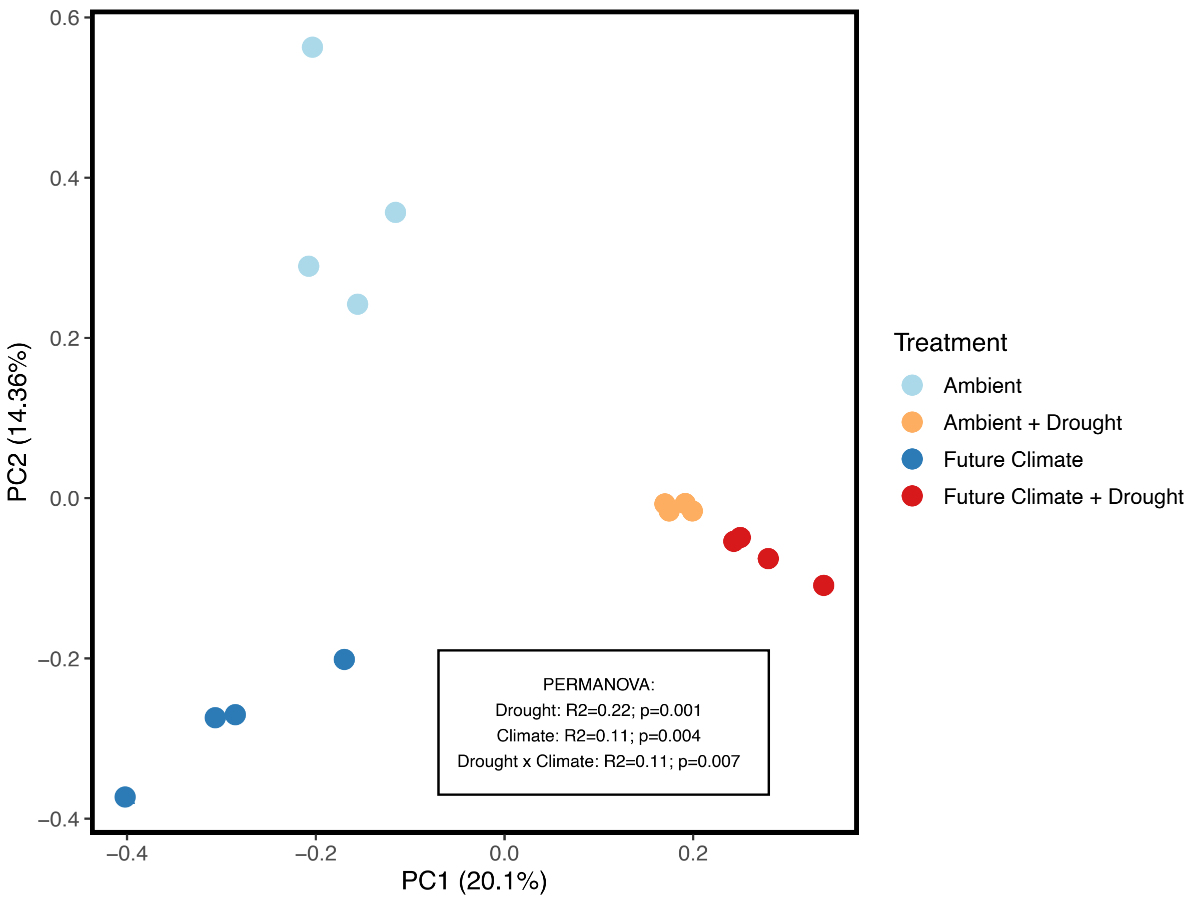

Supplementary Figure 1: Effect of drought and future climate conditions on the growth patterns of bacterial and archaeal communities. Principal component analysis (PCA) on taxon-specific relative growth rates (RGR) of the growing fraction of bacterial and archaeal communities (n=4 replicates). Statistics from two-way permutation-based multivariate analysis of variance (PERMANOVA) testing a full two factorial design (Drought $_{Yes}$, Drought $_{No}$, Climate $_{Ambient}$, Climate $_{Future}$) are provided as inset panels. Source data are provided with this paper.

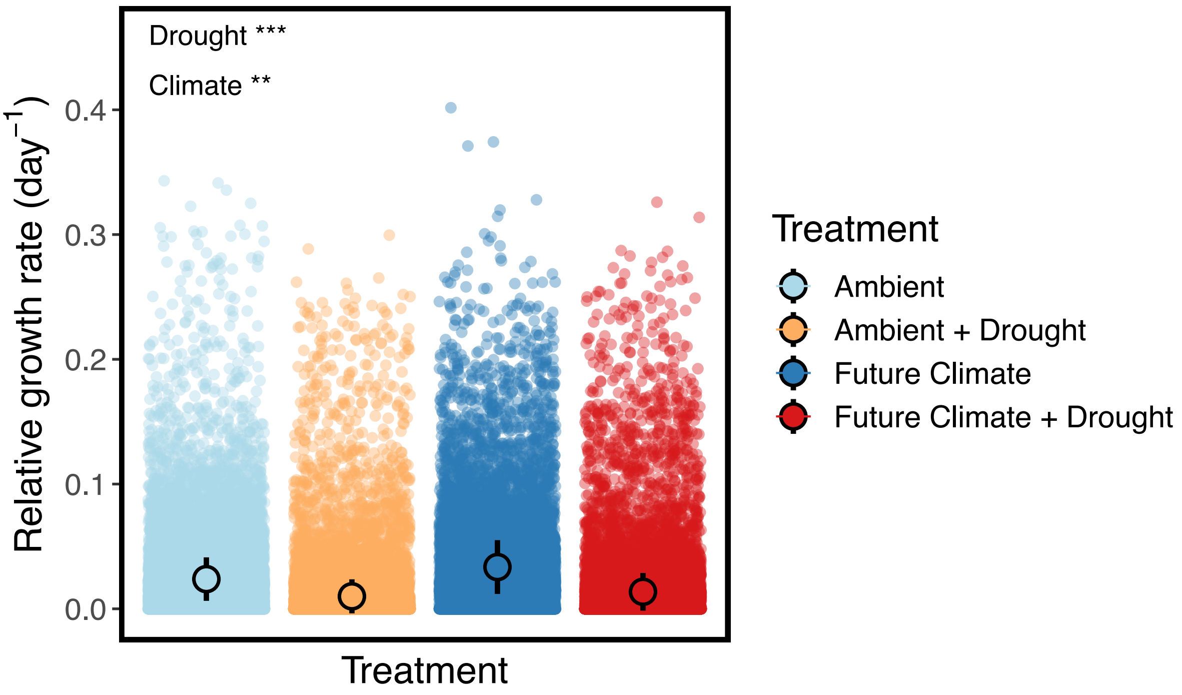

Supplementary Figure 2: Mean taxon-level relative growth rates of bacterial and archaea across treatments including growing and non-growing members. Mean taxon-level relative growth rates (RGR) were calculated using relative growth rates from all taxa that passed prevalence filtering (6054 amplicon sequencing variants (ASVs)). Non-growing taxa as defined per our minimum enrichment threshold (APE $^{18}O > 0.05$) were assigned RGR = 0. Asterisks depict significant results from two-way ANOVA testing a full two factorial design (Drought $_{Yes}$, Drought $_{No}$, Climate $_{Ambient}$, Climate $_{Future}$). Light-colored dots represent ASV-level relative growth rates from all replicates used to calculate means (n=4 means of taxon-specific growth rates calculated per replicate) and standard errors (points, error bars). Source data are provided with this paper.

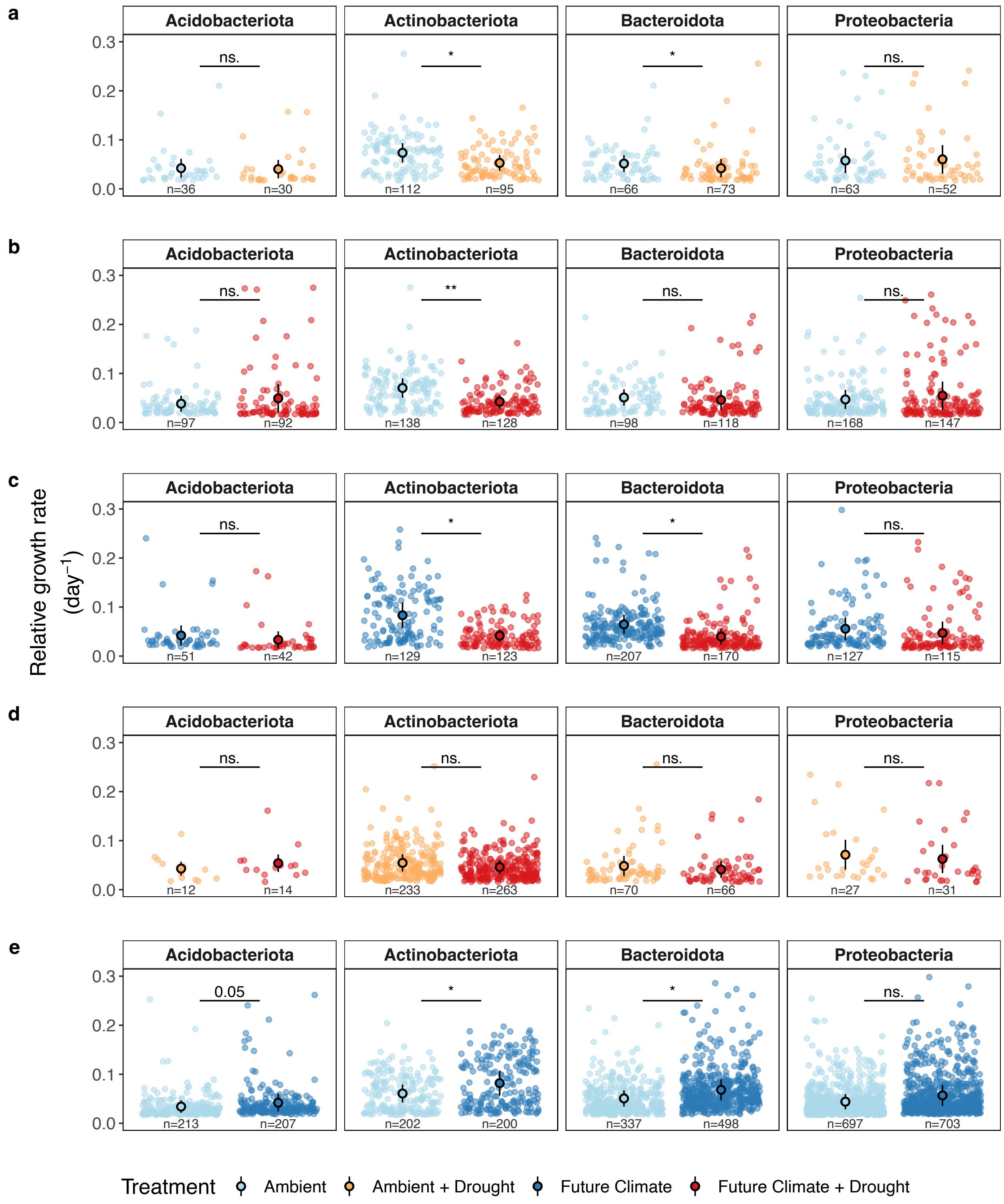
a
b
c
d
e
Acidobacteriota
Actinobacteriota
Bacteroidota
Proteobacteria
Relative growth rate (day^{-1})
0.0
0.1
0.2
0.3
ns.
*
**
0.05
a: n=36 n=30; n=112 n=95; n=66 n=73; n=63 n=52
b: n=97 n=92; n=138 n=128; n=98 n=118; n=168 n=147
c: n=51 n=42; n=129 n=123; n=207 n=170; n=127 n=115
d: n=12 n=14; n=233 n=263; n=70 n=66; n=27 n=31
e: n=213 n=207; n=202 n=200; n=337 n=498; n=697 n=703
Treatment
Ambient
Ambient + Drought
Future Climate
Future Climate + Drought

Supplementary Figure 3: Phylum-specific growth differences between treatments. Phylum-resolved comparison of mean taxon-level relative growth rates of shared ASVs between five treatment pairs (a-e). ASVs belonged to the phyla Acidobacteriota, Actinobacteriota, Bacteroidota, and Proteobacteria. Light-colored dots represent ASV-level growth rates of all replicates used to calculate means and standard errors (points, error bars). P-values of pairwise comparisons are given as insert text. When data fulfilled normality and homoscedasticity requirements, two-sided Student's t-tests were performed. If these criteria were not met, two-sided Wilcoxon tests were performed. The number of actively growing ASVs across all replicates used to calculate mean growth rates (n=4 means of taxon-specific growth rates calculated per replicate) is given below. ASVs had to grow in at least two replicates per treatment to be included. Source data are provided with this paper.

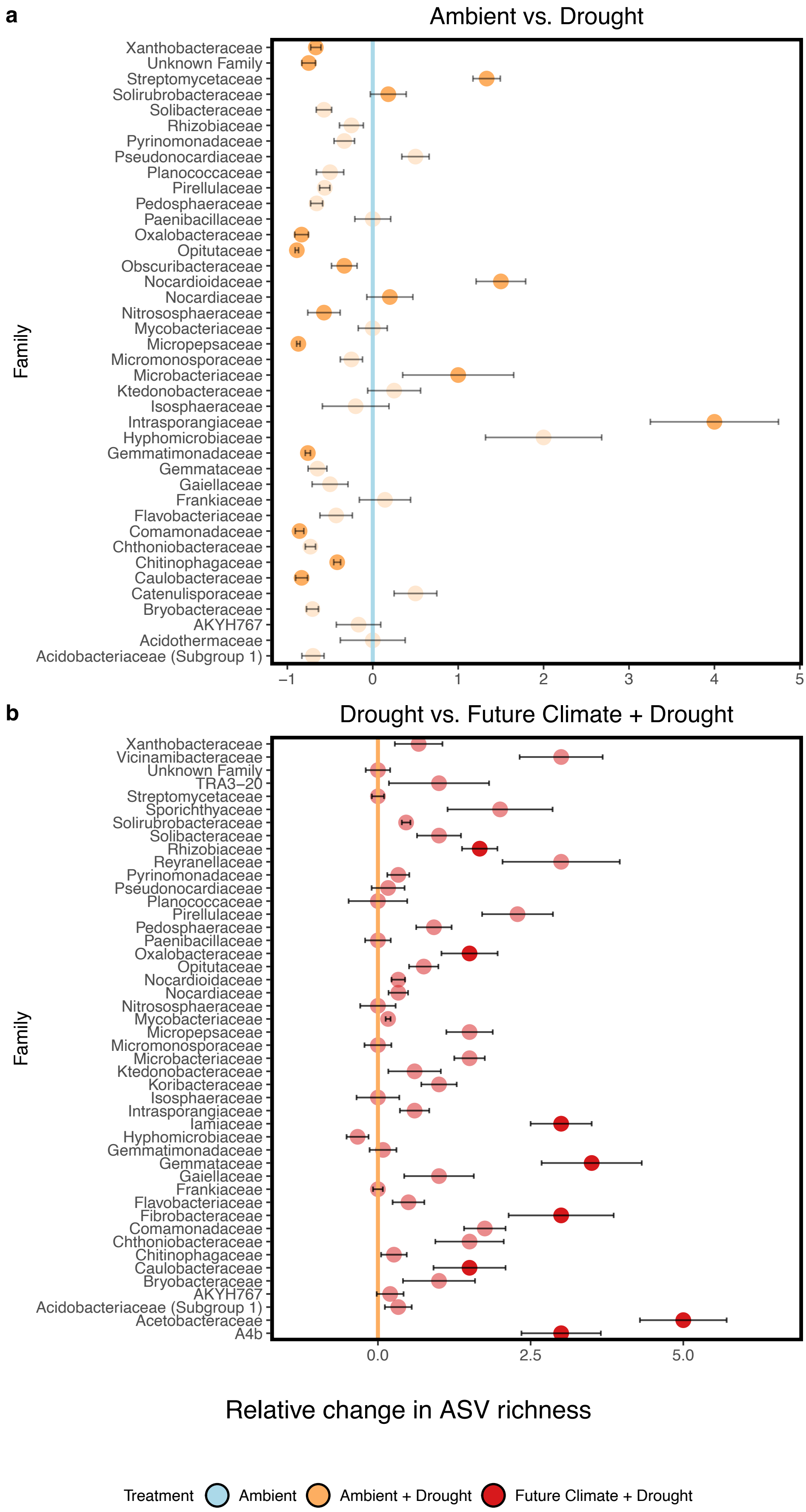
a
Ambient vs. Drought
Family
Xanthobacteraceae
Unknown Family
Streptomycetaceae
Solirubrobacteraceae
Solibacteraceae
Rhizobiaceae
Pyrinomonadaceae
Pseudonocardiaceae
Planococcaceae
Pirellulaceae
Pedosphaeraceae
Paenibacillaceae
Oxalobacteraceae
Opitutaceae
Obscuribacteraceae
Nocardioidaceae
Nocardiaceae
Nitrososphaeraceae
Mycobacteriaceae
Micropepsaceae
Micromonosporaceae
Microbacteriaceae
Ktedonobacteraceae
Isosphaeraceae
Intrasporangiaceae
Hyphomicrobiaceae
Gemmatimonadaceae
Gemmataceae
Gaiellaceae
Frankiaceae
Flavobacteriaceae
Comamonadaceae
Chthoniobacteraceae
Chitinophagaceae
Caulobacteraceae
Catenulisporaceae
Bryobacteraceae
AKYH767
Acidothermaceae
Acidobacteriaceae (Subgroup 1)
−1
0
1
2
3
4
5
b
Drought vs. Future Climate + Drought
Family
Xanthobacteraceae
Vicinamibacteraceae
Unknown Family
TRA3−20
Streptomycetaceae
Sporichthyaceae
Solirubrobacteraceae
Solibacteraceae
Rhizobiaceae
Reyranellaceae
Pyrinomonadaceae
Pseudonocardiaceae
Planococcaceae
Pirellulaceae
Pedosphaeraceae
Paenibacillaceae
Oxalobacteraceae
Opitutaceae
Nocardioidaceae
Nocardiaceae
Nitrososphaeraceae
Mycobacteriaceae
Micropepsaceae
Micromonosporaceae
Microbacteriaceae
Ktedonobacteraceae
Koribacteraceae
Isosphaeraceae
Intrasporangiaceae
Iamiaceae
Hyphomicrobiaceae
Gemmatimonadaceae
Gemmataceae
Gaiellaceae
Frankiaceae
Flavobacteriaceae
Fibrobacteraceae
Comamonadaceae
Chthoniobacteraceae
Chitinophagaceae
Caulobacteraceae
Bryobacteraceae
AKYH767
Acidobacteriaceae (Subgroup 1)
Acetobacteraceae
A4b
0.0
2.5
5.0
Relative change in ASV richness
Treatment
Ambient
Ambient + Drought
Future Climate + Drought

Supplementary Figure 4: Relative changes in the number of growing taxa per family under drought and future drought conditions. **a** Relative change (0 = no change, 1 = increase by 100 %) in the number of growing taxa per family comparing Ambient (reference) and Ambient + Drought conditions. **b** Relative change in the number of growing taxa comparing Ambient + Drought (reference) and Future Climate + Drought conditions. Points represent mean relative changes in the number of growing taxa per family including standard errors (n=4). Two-way ANOVA testing a full two factorial design (Drought $_{Yes}$, Drought $_{No}$, Climate $_{Ambient}$, Climate $_{Future}$) was performed on the number of growing amplicon sequence variants (ASVs) across all treatments for each family independently that fulfilled normality and homoscedasticity requirements. Bold colored points represent significant effects (A: Drought = $p < 0.05$, B: Climate = $p < 0.05$ or Drought x Climate = $p < 0.05$), and transparent points non-significant ones. Vertical lines depict the means of the respective reference treatment used to calculate relative changes. Source data including p-values are provided with this paper.

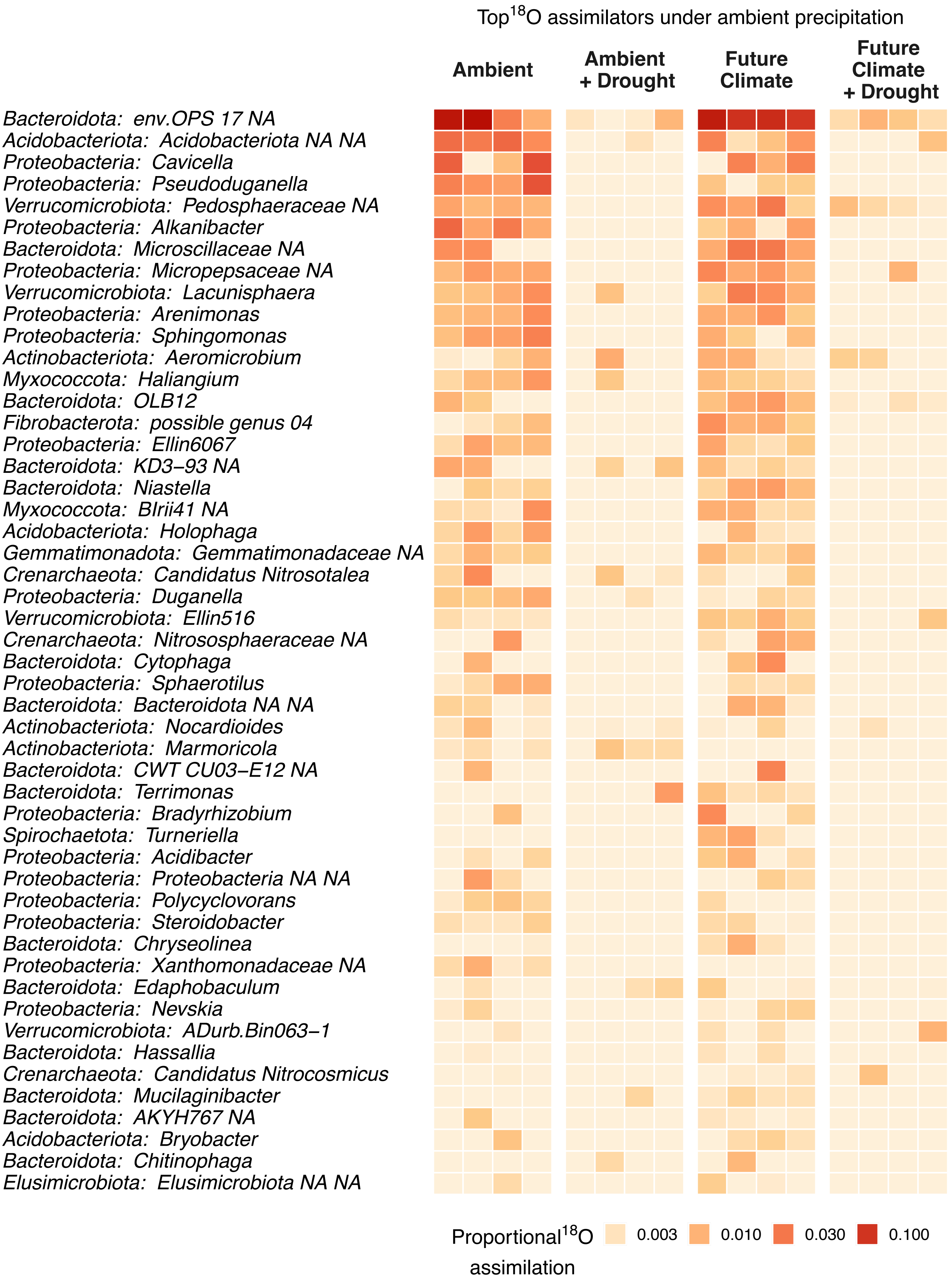

Top^{18}O assimilators under ambient precipitation
Ambient
Ambient + Drought
Future Climate
Future Climate + Drought
Bacteroidota: env.OPS 17 NA
Acidobacteriota: Acidobacteriota NA NA
Proteobacteria: Cavicella
Proteobacteria: Pseudoduganella
Verrucomicrobiota: Pedosphaeraceae NA
Proteobacteria: Alkanibacter
Bacteroidota: Microscillaceae NA
Proteobacteria: Micropepsaceae NA
Verrucomicrobiota: Lacunisphaera
Proteobacteria: Arenimonas
Proteobacteria: Sphingomonas
Actinobacteriota: Aeromicrobium
Myxococcota: Haliangium
Bacteroidota: OLB12
Fibrobacterota: possible genus 04
Proteobacteria: Ellin6067
Bacteroidota: KD3–93 NA
Bacteroidota: Niastella
Myxococcota: Blrii41 NA
Acidobacteriota: Holophaga
Gemmatimonadota: Gemmatimonadaceae NA
Crenarchaeota: Candidatus Nitrosotalea
Proteobacteria: Duganella
Verrucomicrobiota: Ellin516
Crenarchaeota: Nitrososphaeraceae NA
Bacteroidota: Cytophaga
Proteobacteria: Sphaerotilus
Bacteroidota: Bacteroidota NA NA
Actinobacteriota: Nocardioides
Actinobacteriota: Marmoricola
Bacteroidota: CWT CU03–E12 NA
Bacteroidota: Terrimonas
Proteobacteria: Bradyrhizobium
Spirochaetota: Turneriella
Proteobacteria: Acidibacter
Proteobacteria: Proteobacteria NA NA
Proteobacteria: Polycyclovorans
Proteobacteria: Steroidobacter
Bacteroidota: Chryseolinea
Proteobacteria: Xanthomonadaceae NA
Bacteroidota: Edaphobaculum
Proteobacteria: Nevskia
Verrucomicrobiota: ADurb.Bin063–1
Bacteroidota: Hassallia
Crenarchaeota: Candidatus Nitrocosmicus
Bacteroidota: Mucilaginibacter
Bacteroidota: AKYH767 NA
Acidobacteriota: Bryobacter
Bacteroidota: Chitinophaga
Elusimicrobiota: Elusimicrobiota NA NA
Proportional^{18}O assimilation
0.003
0.010
0.030
0.100

Supplementary Figure 5: Top 200 taxa based on their proportional ^{18}O assimilation in soils unaffected by drought, agglomerated at the genus level. Heatmap showing taxa with the highest proportional ^{18}O assimilation under ambient precipitation visualized across all treatments and individual samples (rectangles, n=4). Taxa were ranked based on their proportional ^{18}O assimilation (contribution to the total community's growth) for each drought-unaffected sample (Ambient, Future Climate). The top 50 amplicon sequence variants (ASVs) per sample were then selected (total across all samples due to overlap: 200 ASVS) and visualized. Proportional ^{18}O assimilation ranges from 0 - 1 and estimates how much a single taxon contributes to the community's overall growth. It is calculated using re-computed relative abundances of only growing taxa (sum of growing taxa = 1) and their relative growth rates (RGR). ASVs had to be active in at least two samples if detected as growing in a treatment. ASV identities were agglomerated at the genus level and sorted in descending order based on proportional ^{18}O assimilation. If genus identity could not be assigned (NA), we agglomerated taxa at the family or phylum level. Source data are provided with this paper.

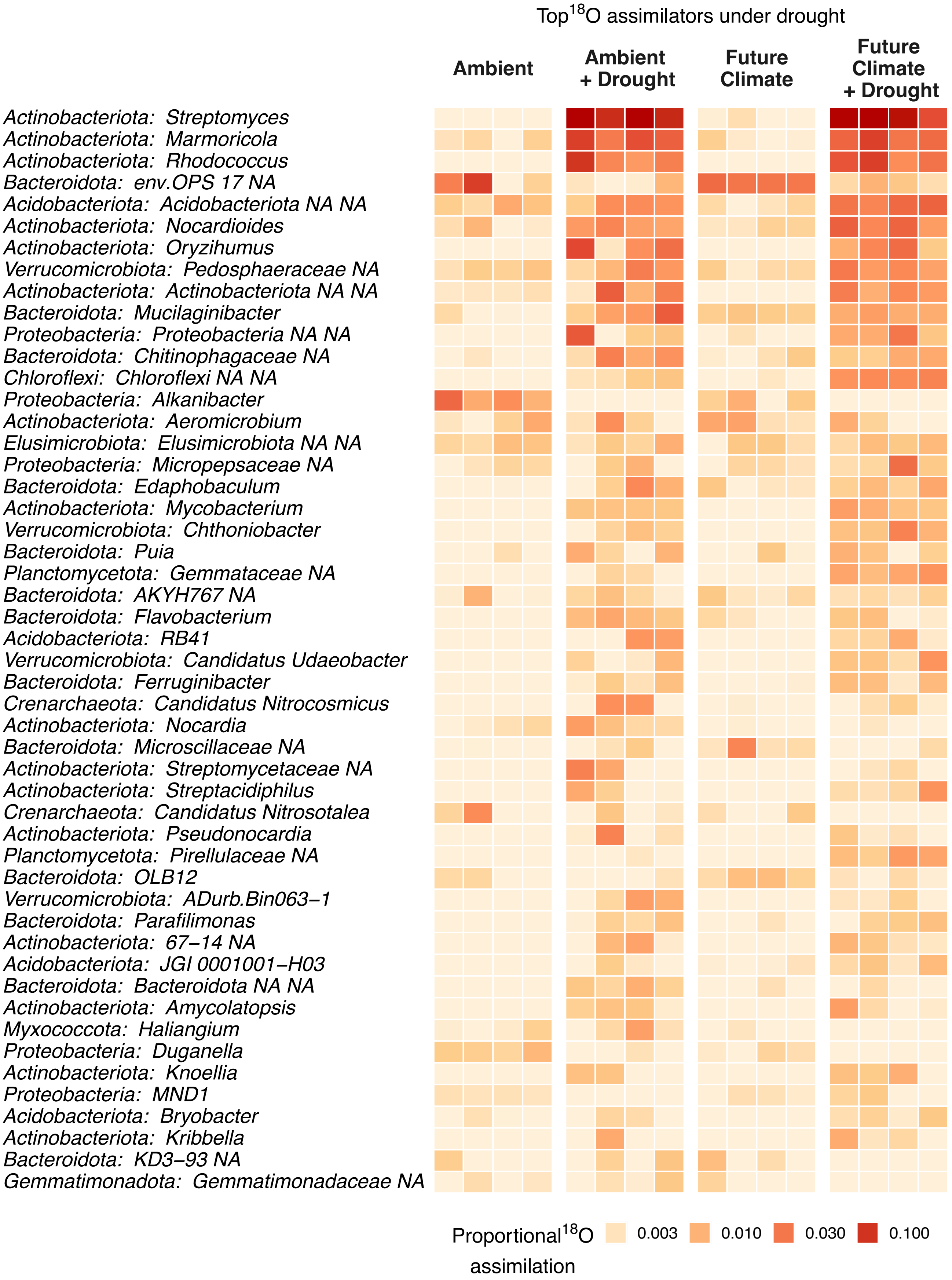
Top^{18}O assimilators under drought
Ambient
Ambient + Drought
Future Climate
Future Climate + Drought
Actinobacteriota: Streptomyces
Actinobacteriota: Marmoricola
Actinobacteriota: Rhodococcus
Bacteroidota: env.OPS 17 NA
Acidobacteriota: Acidobacteriota NA NA
Actinobacteriota: Nocardioides
Actinobacteriota: Oryzihumus
Verrucomicrobiota: Pedosphaeraceae NA
Actinobacteriota: Actinobacteriota NA NA
Bacteroidota: Mucilaginibacter
Proteobacteria: Proteobacteria NA NA
Bacteroidota: Chitinophagaceae NA
Chloroflexi: Chloroflexi NA NA
Proteobacteria: Alkanibacter
Actinobacteriota: Aeromicrobium
Elusimicrobiota: Elusimicrobiota NA NA
Proteobacteria: Micropepsaceae NA
Bacteroidota: Edaphobaculum
Actinobacteriota: Mycobacterium
Verrucomicrobiota: Chthoniobacter
Bacteroidota: Puia
Planctomycetota: Gemmataceae NA
Bacteroidota: AKYH767 NA
Bacteroidota: Flavobacterium
Acidobacteriota: RB41
Verrucomicrobiota: Candidatus Udaeobacter
Bacteroidota: Ferruginibacter
Crenarchaeota: Candidatus Nitrocosmicus
Actinobacteriota: Nocardia
Bacteroidota: Microscillaceae NA
Actinobacteriota: Streptomycetaceae NA
Actinobacteriota: Streptacidiphilus
Crenarchaeota: Candidatus Nitrosotalea
Actinobacteriota: Pseudonocardia
Planctomycetota: Pirellulaceae NA
Bacteroidota: OLB12
Verrucomicrobiota: ADurb.Bin063−1
Bacteroidota: Parafilimonas
Actinobacteriota: 67−14 NA
Acidobacteriota: JGI 0001001−H03
Bacteroidota: Bacteroidota NA NA
Actinobacteriota: Amycolatopsis
Myxococcota: Haliangium
Proteobacteria: Duganella
Actinobacteriota: Knoellia
Proteobacteria: MND1
Acidobacteriota: Bryobacter
Actinobacteriota: Kribbella
Bacteroidota: KD3−93 NA
Gemmatimonadota: Gemmatimonadaceae NA
Proportional^{18}O assimilation
0.003
0.010
0.030
0.100

Supplementary Figure 6: Top 217 taxa based on their proportional ^{18}O assimilation in drought-affected soils, agglomerated at the genus level. Heatmap showing taxa with the highest proportional ^{18}O assimilation under drought visualized across all treatments and individual samples (rectangles, n=4). Taxa were ranked based on their proportional ^{18}O assimilation (contribution to the total community's growth) for each drought-affected sample (Ambient + Drought, Future Climate + Drought). The top 50 amplicon sequence variants (ASVs) per sample were then selected (total across all samples due to overlap: 217 ASVS) and visualized. Proportional ^{18}O assimilation ranges from 0 - 1 and estimates how much a single taxon contributes to the community's overall growth. It is calculated using re-computed relative abundances of only growing taxa (sum of growing taxa = 1) and their relative growth rates (RGR). ASVs had to be active in at least two samples if detected as growing in a treatment. ASV identities were agglomerated at the genus level and sorted in descending order based on proportional ^{18}O assimilation. If genus identity could not be assigned (NA), we agglomerated taxa at the family or phylum level. Source data are provided with this paper.

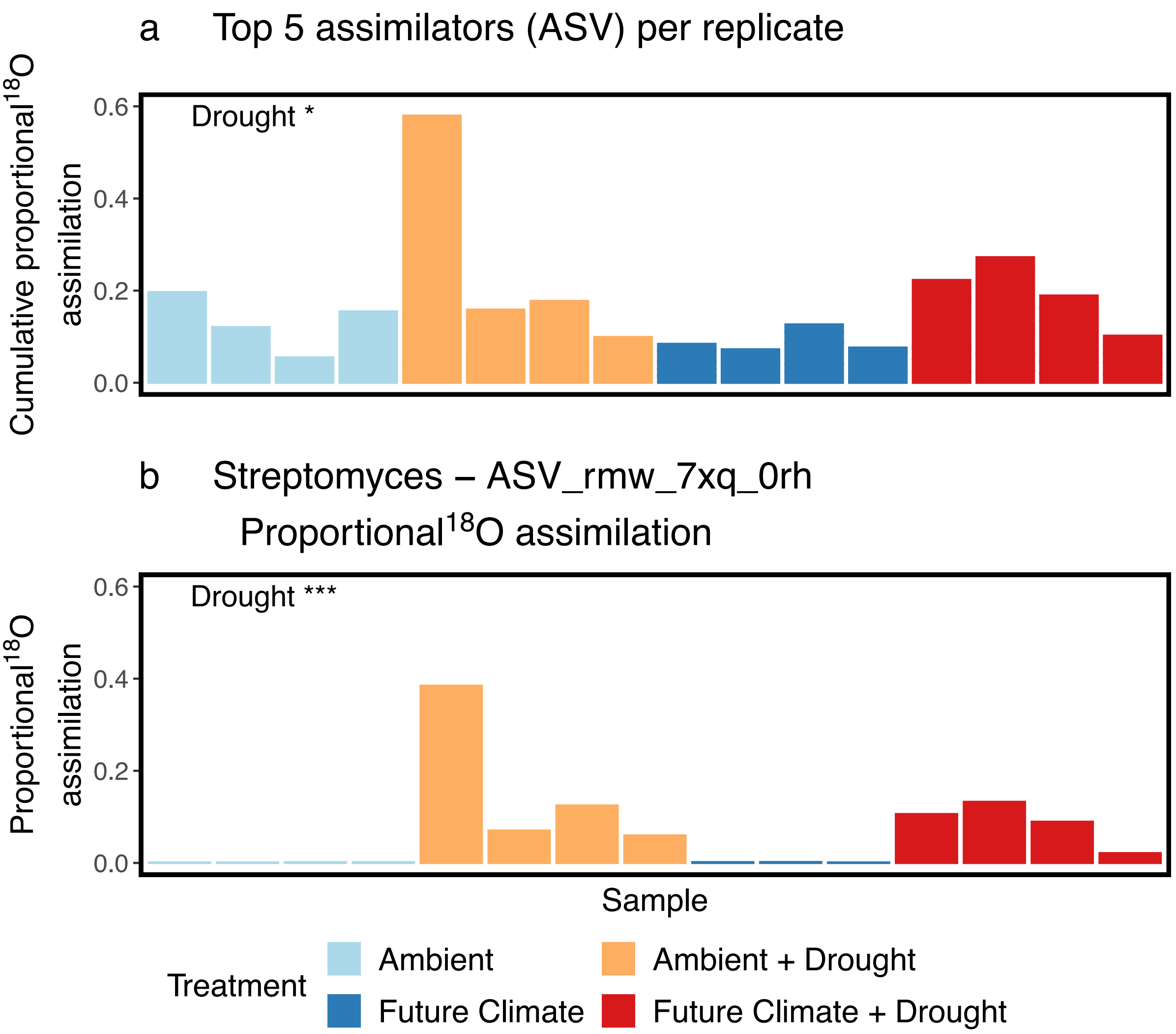

Supplementary Figure 7: Contribution of the top five assimilators and a drought-resistant taxon of the genus *Streptomyces* to the overall community growth. Cumulative proportional ^{18}O assimilation across all samples, calculated as the sum of the proportional ^{18}O assimilation of the top five amplicon sequence variants (ASVs) per replicate (a; Drought: $F = 7.3$, $df = 1$, $p = 0.019$) and ASV_rmw_7xq_0rh (Genus: *Streptomyces*; b; Drought: $F = 159.9$, $df = 1$, $p < 0.001$). Results of two-way ANOVA (n=4 replicates) are given as insert text (factors: Drought $_{Yes}$, Drought $_{No}$, Climate $_{Ambient}$, Climate $_{Future}$). Source data are provided with this paper.

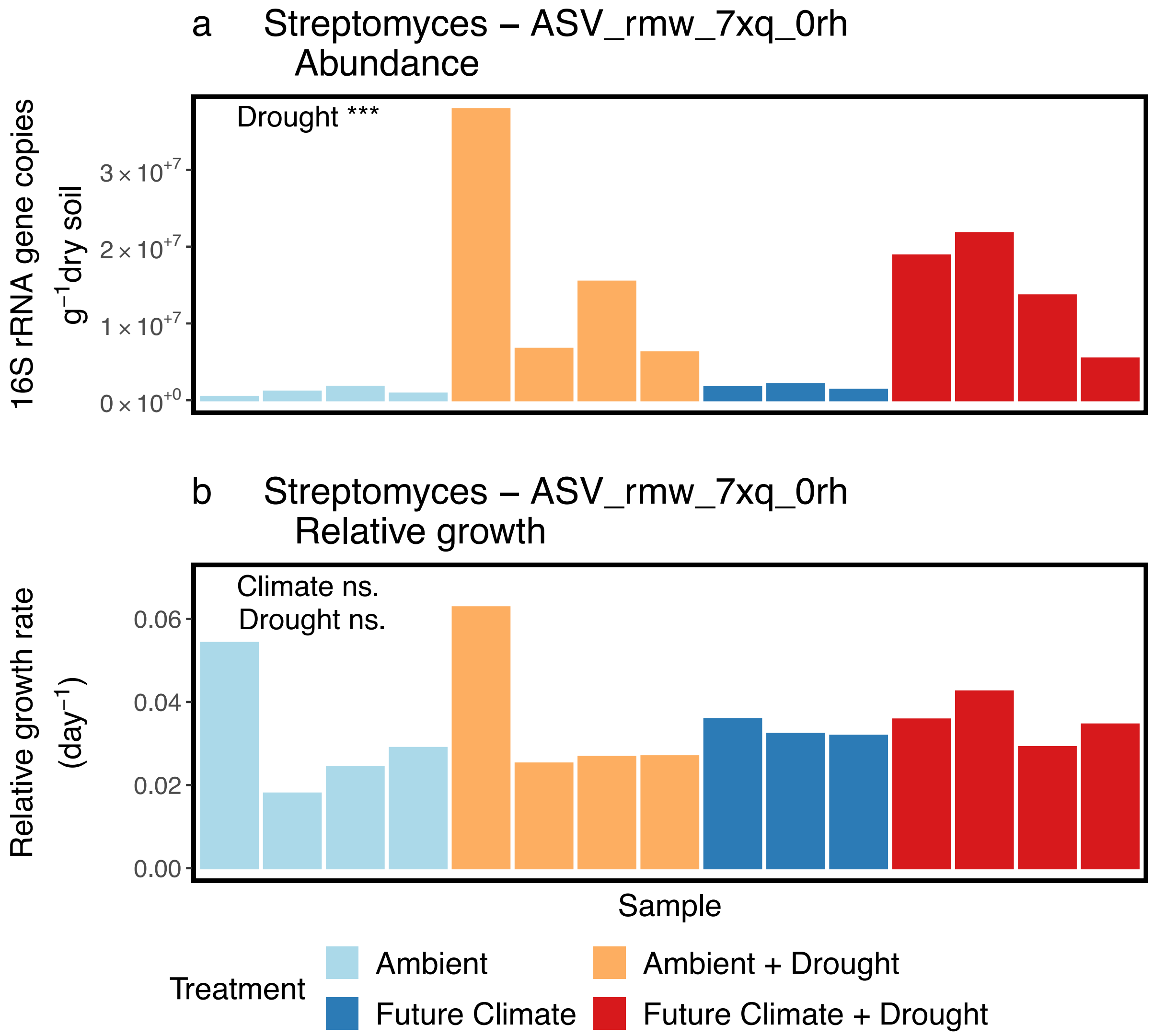

Supplementary Figure 8: Abundance and relative growth of a drought-resistant taxon of the genus *Streptomyces*. a Absolute abundance of ASV_rmw_7xq_0rh (Genus: *Streptomyces*) expressed as 16S rRNA gene copies per gram soil dry weight (Drought: F = 52.4, df = 1, p < 0.001). **b** Relative growth rates of ASV_rmw_7xq_0rh (Genus: *Streptomyces*). Results of two-way ANOVA (n=4 replicates) are given as insert text (factors: Drought $_{Yes}$, Drought $_{No}$, Climate $_{Ambient}$, Climate $_{Future}$). Source data are provided with this paper.

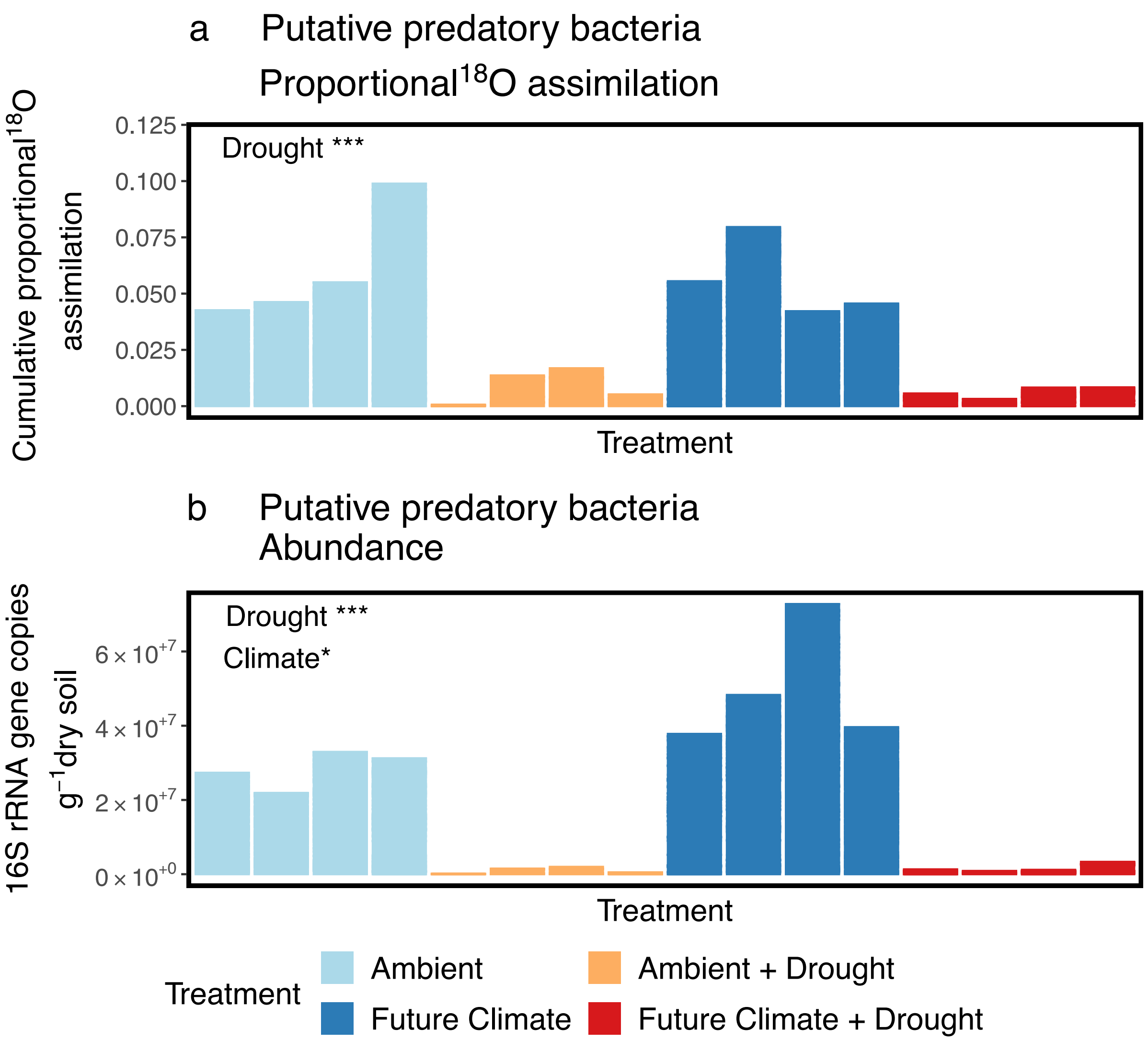

Supplementary Figure 9: Proportional ^{18}O assimilation and abundance of putative predatory bacteria. a Cumulative proportional ^{18}O assimilation across all samples, calculated as the sum of the proportional ^{18}O assimilation of putative predatory amplicon sequence variants (ASVs) per replicate (Drought: F = 63.4, df = 1, $p < 0.001$). **b** Absolute abundance of growing putative predatory ASVs expressed as 16S rRNA gene copies per gram soil dry weight (Drought: F = 227.9, df = 1, $p < 0.001$; Climate: F = 8.9, df = 1, p = 0.01). Results of two-way ANOVA (n=4 replicates) are given as insert text (factors: Drought $_{Yes}$, Drought $_{No}$, Climate $_{Ambient}$, Climate $_{Future}$). Source data are provided with this paper.

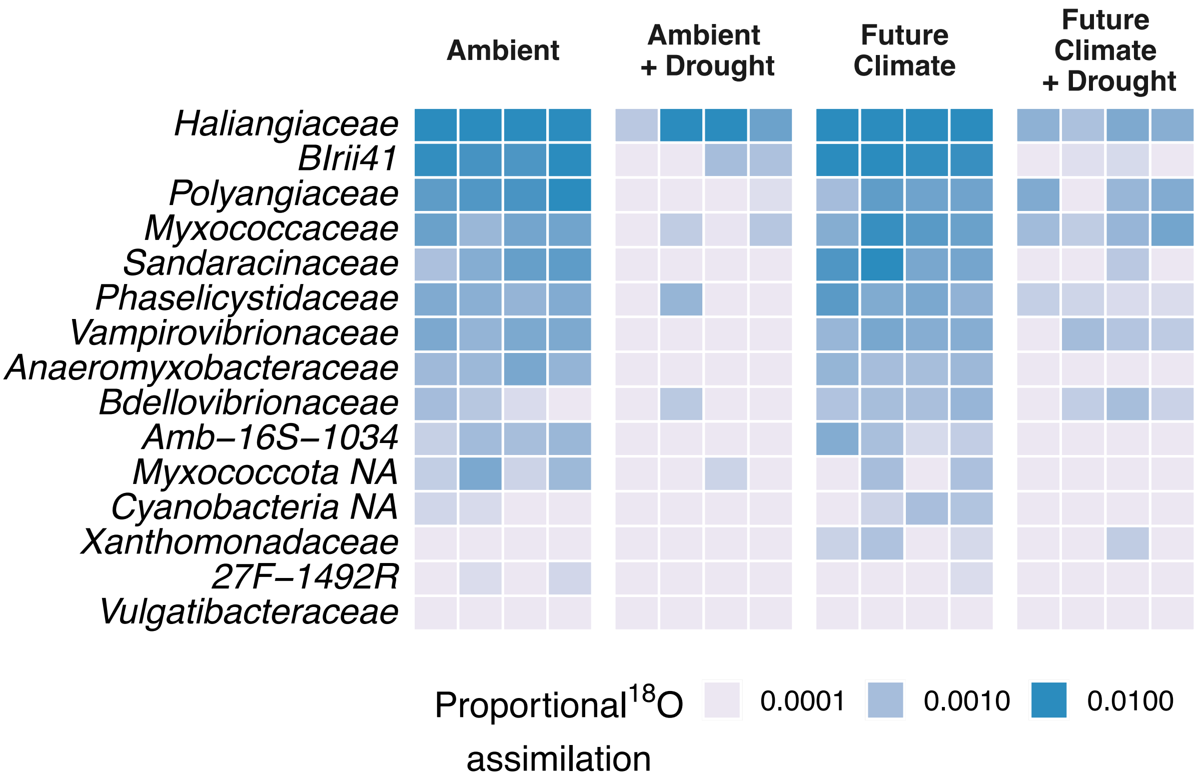

Supplementary Figure 10: Drought-induced changes in the proportional ^{18}O assimilation of putative bacterial predators agglomerated at the family level. Heatmap showing the proportional ^{18}O assimilation of putatively predatory taxa agglomerated at the family level and visualized across all treatments and individual samples (rectangles, n=4 replicates). Taxa were classified as putatively predatory if they belonged to the following orders and genera: Myxococcales, Bdellovibrionales, Vampirovibrionales, Haliangiales, Polyangiales, *Lysobacter, Daptobacter*, and *Vampirococcus*. Proportional ^{18}O assimilation ranges from 0 - 1 and estimates how much a single taxon contributes to the community's overall growth. It is calculated using re-computed relative abundances of only growing taxa (sum of growing taxa = 1) and their relative growth rates (RGR). If family identity could not be assigned (NA), phylum names were provided. Source data are provided with this paper.

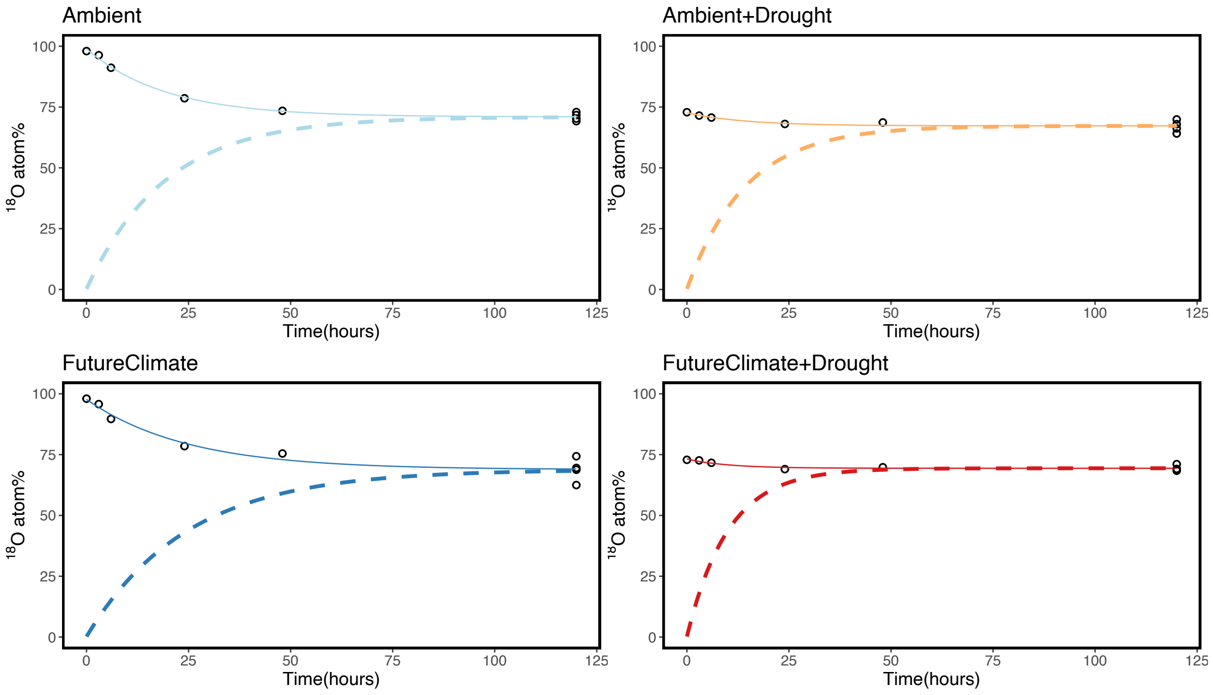

Supplementary Figure 11: Development of ^{18}O soil water enrichment over incubation time. Isotopic equilibration between the externally added ^{18}O labeled source water (solid line) and soil water (dashed line) over five days for all four treatments. Points represent measured ^{18}O atom% values of the added water pool. Data were fitted using a negative exponential model (solid line) and used to predict ^{18}O enrichment of the soil water. Source data are provided with this paper.
