# Supplementary material for: Microbial growth under drought is confined to distinct taxa and modified by potential future climate conditions
Source: Nat Commun. 2023 Sep 22;14:5895. doi: 10.1038/s41467-023-41524-y (PMC10516970; doi:10.1038/s41467-023-41524-y)
Supplement: Supplementary file 3 — Reporting Summary [file 41467_2023_41524_MOESM3_ESM.pdf]

nature portfolio

Corresponding author(s): Dennis Metze

Last updated by author(s): Aug 28, 2023

# Reporting Summary

Nature Portfolio wishes to improve the reproducibility of the work that we publish. This form provides structure for consistency and transparency in reporting. For further information on Nature Portfolio policies, see our Editorial Policies and the Editorial Policy Checklist.

## Statistics

For all statistical analyses, confirm that the following items are present in the figure legend, table legend, main text, or Methods section.

| n/a | Confirmed | |
|---|---|---|
| ☐ | ☒ | The exact sample size (*n*) for each experimental group/condition, given as a discrete number and unit of measurement |
| ☐ | ☒ | A statement on whether measurements were taken from distinct samples or whether the same sample was measured repeatedly |
| ☐ | ☒ | The statistical test(s) used AND whether they are one- or two-sided<br>*Only common tests should be described solely by name; describe more complex techniques in the Methods section.* |
| ☒ | ☐ | A description of all covariates tested |
| ☐ | ☒ | A description of any assumptions or corrections, such as tests of normality and adjustment for multiple comparisons |
| ☐ | ☒ | A full description of the statistical parameters including central tendency (e.g. means) or other basic estimates (e.g. regression coefficient) AND variation (e.g. standard deviation) or associated estimates of uncertainty (e.g. confidence intervals) |
| ☐ | ☒ | For null hypothesis testing, the test statistic (e.g. *F*, *t*, *r*) with confidence intervals, effect sizes, degrees of freedom and *P* value noted<br>*Give P values as exact values whenever suitable.* |
| ☒ | ☐ | For Bayesian analysis, information on the choice of priors and Markov chain Monte Carlo settings |
| ☒ | ☐ | For hierarchical and complex designs, identification of the appropriate level for tests and full reporting of outcomes |
| ☒ | ☐ | Estimates of effect sizes (e.g. Cohen's *d*, Pearson's *r*), indicating how they were calculated |

*Our web collection on statistics for biologists contains articles on many of the points above.*

## Software and code

Policy information about availability of computer code

| | |
|---|---|
| Data collection | No software was used to collect the data |
| Data analysis | The R software (version 4.1.1) was used for statistical analyses and figures. To calculate taxon-specific 18O atom percent excess (APE) we used publicly available code (https://bitbucket.org/QuantitativeSIP/qsip_repo, https://github.com/bramstone/qsip). We used 'ggplot2' (version 3.3.6) and 'ampvis2' (version 2.7.11) for graphs. We used the 'aov' function to test the two-way ANOVA, the 'p.adjust' function for the FDR correction, the "pairwise.wilcox.test" for pairwise Wilcoxon signed-rank tests, and the "lmer" function from the "lme4" (version '1.1.27.1') package to perform for linear mixed effect models, and the ''adonis' function to conduct two-way PERMANOVA. We used the 'phyloseq' (version 1.38.0) package to manipulate amplicon sequencing data. To manipulate data tables, we used the packages 'reshape' (version 1.4.4) and 'data.table' (version 1.14.2). We performed principal component analysis using the ´prcomp´ ('stats' ,version 4.1.1) and ´autoplot´ ('ggplot2' ,version 3.3.6) functions.We removed contaminant ASVs (ASVs) identified using the 'decontam' package (version 1.6.0 58). Demultiplexing of amplicon pools was performed with the python package demultiplex (Laros JFJ, github.com/jfjlaros/demultiplex). Amplicon sequence variants were inferred using the 'DADA2' package (version 1.20). Software usage is described in detail in the Methods section. All custom code and data used to produce the analyses and figures of this study are openly available in the Zenodo database under accession code 8109566 [http://doi.org/10.5281/zenodo.8109566.] |

For manuscripts utilizing custom algorithms or software that are central to the research but not yet described in published literature, software must be made available to editors and reviewers. We strongly encourage code deposition in a community repository (e.g. GitHub). See the Nature Portfolio guidelines for submitting code & software for further information.

## Data

Policy information about availability of data

All manuscripts must include a data availability statement. This statement should provide the following information, where applicable:

- Accession codes, unique identifiers, or web links for publicly available datasets
- A description of any restrictions on data availability
- For clinical datasets or third party data, please ensure that the statement adheres to our policy

16S-V4 regions were classified using the SILVA SSU database (Ref NR 99 release 138.1). The sequencing data generated in this study including all metadata crucial to perform all analyses have been deposited in the NCBI Sequence Read Archive under BioProject accession number PRJNA937073.All data used to produce the analyses and figures of this study are openly available in the Zenodo database under accession code 8109566 [http://doi.org/10.5281/zenodo.8109566.]

## Human research participants

Policy information about studies involving human research participants and Sex and Gender in Research.

| | |
|---|---|
| Reporting on sex and gender | N/ A |
| Population characteristics | N/ A |
| Recruitment | N/ A |
| Ethics oversight | N/ A |

Note that full information on the approval of the study protocol must also be provided in the manuscript.

# Field-specific reporting

Please select the one below that is the best fit for your research. If you are not sure, read the appropriate sections before making your selection.

☐ Life sciences ☐ Behavioural & social sciences ☒ Ecological, evolutionary & environmental sciences

For a reference copy of the document with all sections, see nature.com/documents/nr-reporting-summary-flat.pdf

# Ecological, evolutionary & environmental sciences study design

All studies must disclose on these points even when the disclosure is negative.

**Study description**

This field experiment explored the effects of drought, future climate conditions (+ 3°C of warming, + 300ppm of atmospheric CO2), and their combination on the growing community of soil bacteria and archaea and their taxon-specific growth rates. From 2014 onwards future climate conditions were simulated using infrared heaters and miniFACE systems. Severe summer drought was simulated using automated rainout shelters installed for six weeks (June 17th – August 3rd, 2020). Samples were collected from all four replicate plots of four treatments according to a full two-factorial design: (i) ambient conditions, (ii) ambient conditions & drought, (iii) future climate conditions (+3 °C and +300 ppm), and (iv) future climate conditions & drought.
Soil samples were taken at the end of the drought simulation and immediately transported to the laboratory. Samples were sieved and immediately used to measure water content, prior to qSIP incubations with 18O-H2O (5 days) to estimate taxon-specific growth rates. We used the water vapor equilibration method to enrich soil water with 18O-H2O. At the end of the incubations, samples were snap-frozen in liquid nitrogen and stored at -80°C until DNA extraction and ultracentrifugation. After DNA precipitation, DNA was stored at -20°C and used for 16S rRNA gene quantification using digital droplet PCR.

**Research sample**

Topsoil samples were collected using a soil core (10 cm deep, 2 cm diameter) to probe soil microbial communities and passed through a 2 mm sieve. The topsoil was chosen since it is the most active soil horizon that is responsible for the majority of microbial activity and associated biogeochemical cycling.

**Sampling strategy**

For our study, we made use of the ClimGrass experiment located in a managed grassland in Central Austria. ClimGrass is a multifactorial climate change experiment fully operational since 2014, comprising 54 experimental plots (4m x 4m) and six treatment conditions characterized by joint or individual manipulations of temperature (ambient, +1.5 °C, + 3 °C), atmospheric CO2 (ambient, +150ppm, +300 ppm), and severe summer drought. We selected four treatments according to a full two-factorial design ((i) ambient conditions, (ii) ambient conditions & drought, (iii) future climate conditions (+3 °C and +300 ppm), and (iv) future climate conditions & drought) to assess single and interactive effects of drought and future climate conditions. We sampled all four replicate plots per treatment after the simulation of a severe summer drought event in 2020.

**Data collection**

Data such as soil weights, soil water content, and soil temperature were collected manually using precision balances, a drying oven, and a soil temperature thermometer by Alberto Canarini, Lucia Fuchslueger, and Jörg Schnecker. To model the temporal dynamics of

| | |
|---|---|
| | the isotopic equilibration of the soil water during laboratory incubations with 18O water, we collected the remains of the previously added labelled water and analyzed it for its isotopic composition through equilibration of 18O in H2O with CO2 on a Gasbench II headspace sampler connected to a Delta V Advantage isotope ratio mass spectrometer. DNA concentrations were quantified fluorometrically using the PicoGreen assay. The density of each fraction was determined with a Krüss DR301-95 digital refractometer. Amplicon sequencing was performed on a Illumina MiSeq (V3 Kit) in the 2 x 300 bp configuration and concentrations of archaeal and bacterial 16S rRNA gene copies were quantified using the Bio-Rad QX200 Droplet Digital PCR (ddPCR) system. |
| Timing and spatial scale | The managed grassland (47°29′38′′N, 14°06′03′′E, 710m, MAT: 8.2 °C, MAT: 1056 mm) represents a typical mountain grassland of many parts of the Alps. The ClimGrass experiment follows a response-surface design with 54 plots (4m x 4m). Harvesting is restricted to an area of 1m x 1m. Heating is applied all day and all year round unless the snow cover exceeds a continuous height of 10 cm. All samples were collected on July 29th , 2020 at peak summer drought after 6 weeks of drought simulation in the drought-affected plots. Samples were transported to the University of Vienna on the day of harvest and sieved. After determining the soil water content, samples were incubated for 5 days and snap-frozen after incubation. Subsequent steps including DNA extraction, ultracentrifugation, and ddPCR were performed at the University of Vienna in the following year. |
| Data exclusions | No data was excluded from the analysis. |
| Reproducibility | No attempts have been made to reproduce the experiment but a detailed description of the laboratory methods and used code allow for reproduction. Though, the timing and conditions of unmanipulated environmental conditions inherent to the field experiment are impossible to reproduce. |
| Randomization | The ClimGrass experiment follows a response-surface design with 54 plots (4m x 4m).<br>Treatments were allocated randomly across the experimental field and a subset of the treatments was used for the current study. |
| Blinding | Blinding was not done since samples were taken from specific treatments and had to be treated according to their treatment for lab incubations. |

Did the study involve field work? ☒ Yes ☐ No

## Field work, collection and transport

| | |
|---|---|
| Field conditions | The climatic site conditions at the field site were characterized by a mean annual temperature of 8.2°C and a mean annual precipitation of 1056 mm. The soil type is a Cambisol with a pH of ~5.5. All plots are fertilized with mineral fertilizer due to nutrient removal induced by harvests. |
| Location | The field site is situated in managed grassland at the Agricultural Research and Education Centre (AREC) Raumberg-Gumpenstein (Austria, 47°29′38′′N, 14°06′03′′E). |
| Access & import/export | All plots were accessible by car/walking and we were accompanied by local scientists or employees working for the Agricultural Research and Education Centre (AREC) Raumberg-Gumpenstein. Soil samples were all collected within Austria and no permits were necessary for transportation. |
| Disturbance | When the experiment was constructed, disturbance was not avoidable. To reduce disturbance on the plots at the time of sampling only one person was allowed to collect soil samples. |

# Reporting for specific materials, systems and methods

We require information from authors about some types of materials, experimental systems and methods used in many studies. Here, indicate whether each material, system or method listed is relevant to your study. If you are not sure if a list item applies to your research, read the appropriate section before selecting a response.

### Materials & experimental systems

| n/a | Involved in the study |
|---|---|
| ☒ | ☐ Antibodies |
| ☒ | ☐ Eukaryotic cell lines |
| ☒ | ☐ Palaeontology and archaeology |
| ☒ | ☐ Animals and other organisms |
| ☒ | ☐ Clinical data |
| ☒ | ☐ Dual use research of concern |

### Methods

| n/a | Involved in the study |
|---|---|
| ☒ | ☐ ChIP-seq |
| ☒ | ☐ Flow cytometry |
| ☒ | ☐ MRI-based neuroimaging |
